# Stat3 orchestrates interaction between endothelial and tumor cells and inhibition of Stat3 suppresses brain metastasis of breast cancer cells

**DOI:** 10.18632/oncotarget.3540

**Published:** 2015-03-12

**Authors:** Hsueh-Te Lee, Jianfei Xue, Ping-Chieh Chou, Aidong Zhou, Phillip Yang, Charles A. Conrad, Kenneth D. Aldape, Waldemar Priebe, Cam Patterson, Raymond Sawaya, Keping Xie, Suyun Huang

**Affiliations:** ^1^ Department of Neurosurgery, The University of Texas MD Anderson Cancer Center, Houston, Texas, USA; ^2^ Department of Neuro-Oncology, The University of Texas MD Anderson Cancer Center, Houston, Texas, USA; ^3^ Department of Pathology, The University of Texas MD Anderson Cancer Center, Houston, Texas, USA; ^4^ Department of Experimental Therapeutics, The University of Texas MD Anderson Cancer Center, Houston, Texas, USA; ^5^ Institute of Anatomy and Cell Biology, National Yang-Ming University, Taipei, Taiwan; ^6^ Division of Cardiology and McAllister Heart Institute, University of North Carolina, Chapel Hill, North Carolina, USA; ^7^ Gastroenterology, Hepatology & Nutrition, The University of Texas MD Anderson Cancer Center, Houston, Texas, USA; ^8^ Program in Cancer Biology, The University of Texas Graduate School of Biomedical Sciences at Houston, Houston, Texas, USA

**Keywords:** Stat3, VEGFR2, Stat3 inhibitor, brain metastasis, brain endothelial cells

## Abstract

Brain metastasis is a major cause of morbidity and mortality in patients with breast cancer. Our previous studies indicated that Stat3 plays an important role in brain metastasis. Here, we present evidence that Stat3 functions at the level of the microenvironment of brain metastases. Stat3 controlled constitutive and inducible VEGFR2 expression in tumor-associated brain endothelial cells. Furthermore, inhibition of Stat3 by WP1066 decreased the incidence of brain metastases and increased survival in a preclinical model of breast cancer brain metastasis. WP1066 inhibited Stat3 activation in tumor-associated endothelial cells, reducing their infiltration and angiogenesis. WP1066 also inhibited breast cancer cell invasion. Our results indicate that WP1066 can inhibit tumor angiogenesis and brain metastasis mediated by Stat3 in endothelial and tumor cells.

## INTRODUCTION

The major cause of death from breast cancer is metastases that are resistant to conventional therapies [1]. Brain metastasis occurs in about 20% of patients with breast cancer and is a major cause of death in patients with breast cancer [2]. The prognosis of patients with brain metastasis is poor [3-5], with a median survival of 4 to 6 months, because brain metastases are largely refractory to current treatments, including surgery, radiation therapy, and chemotherapy. Thus, targeted treatment and prevention strategies for brain metastasis are needed to improve the survival rate of breast cancer patients.

The formation of brain metastases is determined by both tumor cell properties and host factors. Tumor cells may release vascular endothelial growth factor (VEGF; also known as vascular permeability factor), inducing neovascularization in brain metastases [6]. Indeed, the progressive growth of brain metastases is dependent on expression of VEGF. VEGF activates signaling in endothelial cells after binding receptors on the cell surface, including VEGF receptor 2 (VEGFR2) (KDR/Flk-1). VEGFR2 signaling activates a variety of downstream signaling pathways in endothelial cells, including the Stat3, PI3K, and MEK-ERK pathways [7-9], and is responsible for many of the characteristic effects of VEGF on endothelial cells, including cell proliferation, survival, migration, and increased vascular permeability [10-12]. Moreover, VEGF induces expression of its receptors, including VEGFR2 [13, 14], but its molecular mechanisms are not fully understood. In the present study, we found Stat3 is a direct transcription factor for VEGFR2 and increases its expression.

Stat3 is a member of the JAK-STAT (Janus kinase-signal transducer and activator of transcription) signaling pathway [15, 16]. In breast cancer cells, both genetic and epigenetic alterations have been shown to activate Stat3, including alterations in Her2/Neu, EGFR, BRCA1, and estrogen receptor [17, 18]. Overexpression of constitutively activated Stat3 in immortalized breast cells induced tumor formation [19]. Stat3 has also been linked with cellular proliferation, promotion of apoptosis, invasion, angiogenesis, and evasion of host immune responses [20-25]. Furthermore, Stat3 activation has been implicated as an important driver of brain metastasis in breast cancer, although the mechanisms are not fully understood [26, 27].

In the current study, we sought to determine the role of Stat3 in the interaction between endothelial cells and tumor cells in breast cancer brain metastasis. We tested the effect of a Stat3 inhibitor, WP1066 [29-31], on breast cancer brain metastasis in a preclinical mouse model. WP1066 has been shown to effectively block JAK2 and Stat3 activation in multiple tumor cell types, including glioma cell lines [29, 30]. We also explored the mechanisms underlying the inhibitory effects of WP1066 on metastasis of breast cancer to the brain. We demonstrate that WP1066 reduced the incidence of brain metastasis and significantly extended the overall survival of metastasis-bearing mice. We also show a unique role of Stat3 activation via VEGFR2 in orchestrating the interaction between endothelial and brain tumor cells and promoting breast cancer brain metastasis.

## RESULTS

### Stat3 activation correlated directly with the brain metastatic potential of breast cancer cells

**Figure 1:**
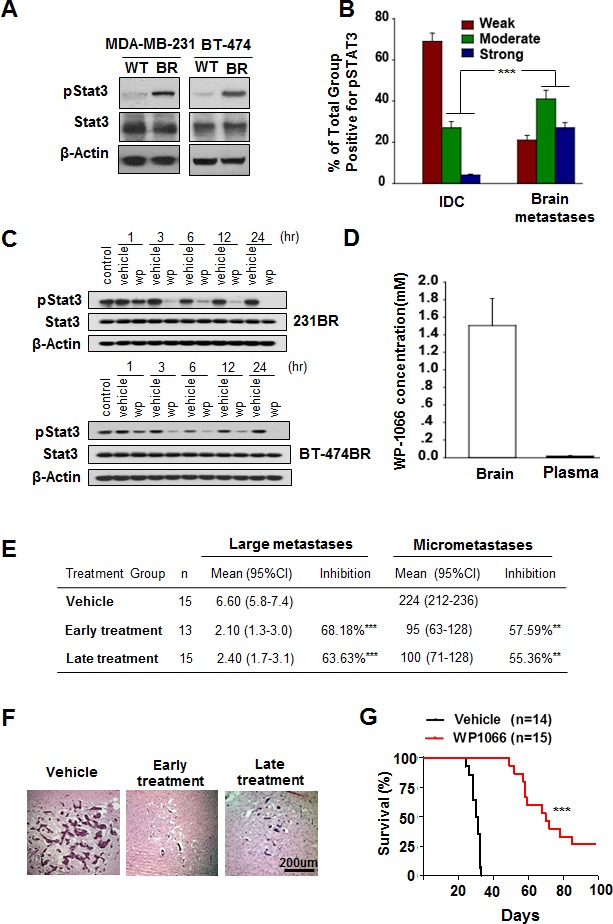
Stat3 activation in breast cancer brain metastases and WP1066 inhibited brain metastasis (A) pStat3, Stat3, and β-actin protein expression levels in MDA-MB-231 and BT-474 cells. (B) Expression levels of pStat3 in 90 IDC and 89 breast cancer brain metastasis specimens. ***, P<0.001. (C) MDA-MB-231BR cells and BT-474BR cells were treated with 1 μM WP1066 (wp) for the indicated times, and whole-cell lysates were subjected to western blotting for pStat3 (Tyr705), Stat3 and β-actin. (D) Concentration of WP1066 in mouse brain tissue and plasma after WP1066 treatment for 72 hours. (E) The effect of WP1066 on the brain metastases of MDA-MB231-BR cell *in vivo.* 5×10^5^ MDA-M-B231-BR cells were injected into the Left ventricle of the heart of nude mice. Results were shown for one representative experiment of two. **, P<0.01, ***, P<0.001. (F) HE-stained sections of brain metastases of MDA-MB-231BR cells in mice. (G) Survival of mice injected with MDA-MB-231BR cells and given later WP1066 treatment. Data are presented from the day of injection to day 100. Survival of mice was evaluated by Kaplan-Meier analysis. ***, P<0.001.

We first examined Stat3 activity in MDA-MB-231 and BT-474 cell lines and in brain metastatic cell lines MDA-MB-231BR and BT-474BR. Stat3 activity is significantly higher in MDA-MB-231BR and BT-474BR cells than in their wild-type counterparts (Fig. 1A), indicating that Stat3 was constitutively activated at higher levels in brain metastatic breast cancer cells.

Next, we analyzed 90 breast invasive ductal carcinoma (IDC) and 89 breast cancer brain metastasis specimens using immunohistochemistry for nuclear staining of pStat3, the activated form of Stat3. 5.5% of the IDC specimens exhibited strong positive, 25.6% moderate positive, and 68.9% weak to negative staining for pStat3 (Fig. 1B and Supplementary Fig. S1). In contrast, 30.3% of the brain metastasis specimens exhibited strong positive, 46.1% moderate positive, and 23.6% weak to negative staining for pStat3. When the data regarding strong positive and moderate positive staining were analyzed using χ^2^ test, significantly higher levels of pStat3 were evident in breast cancer brain metastases than in IDC specimens (Fig. 1B; P < 0.001).

### WP1066 inhibited Stat3 activation in breast cancer brain metastatic cells

On the basis of the above findings, we hypothesized that treatment with WP1066, a Stat3 inhibitor [29], would inhibit brain metastasis by reducing Stat3 activation. MDA-MB-231BR and BT-474BR cells were treated with 1 μM WP1066 for 1 to 24 hours and then examined for levels of pStat3. WP1066 substantially decreased pStat3 level in both cell lines in a time-dependent manner (Fig. 1C; Supplementary Fig. S2).

### Brain permeability of WP1066

To determine the brain permeability of WP1066, WP1066 (40 mg/kg) was injected intraperitoneally into nude mice every other day until three doses had been given. After the third dose, the brains were harvested from mice, and the plasma and brain concentrations of WP1066 were measured by LC/MS/MS. WP1066 distribution into the brain was more favorable than WP1066 distribution into plasma. The concentration of WP1066 in brain tissue was 1.06 μM to 1.81 μM (mean 1.50 μM) (Fig. 1D). In contrast, the concentration of WP1066 in plasma was 0.10 μM to 0.027 μM (mean 0.018 μM) (Fig. 1D). Moreover, the mean brain/plasma ratio of WP1066 was 92.8 (Fig. 1D), indicating that brain concentrations of WP1066 were more than 90 times plasma concentrations. These data indicated a potentially high distribution of WP1066 into brain tissue, suggesting activity of WP1066 against brain metastases.

### WP1066 inhibited brain metastases of breast cancer cells in nude mice

We used the well-established brain metastases model of MDA-MB-231BR cells to test the effect of WP1066 on brain metastases [28]. WP1066 treatment (40 mg/kg) began on day 3 (early treatment) or 9 (late treatment) after tumor cell injection and continued every other day until six doses had been given (Supplementary Fig. 1C). Thirty days after tumor cell injection, the brains were harvested from mice of each group, and the numbers of metastases were determined. Early administration of WP1066 reduced the number of large metastases by 68.18%, and reduced the number of micrometastases by 57.59% (Fig. 1E). Late administration of WP1066 reduced the number of large metastases by 63.63%, and reduced the number of micrometastases by 55.36%.

We also determined the effect of WP1066 on survival of mice bearing brain metastases over a 100-day period. As shown in Fig. 1F and G, MDA-MB-231BR cells produced brain metastases in all of the injected mice, and the mice became moribund around 35 days after cell injection. In contrast, early treatment with WP1066 significantly increased the survival of the mice injected with MDA-MB-231BR cells (*P* <0.001). These results showed that WP1066 treatment suppressed breast cancer cell brain metastasis and increased survival duration in a mouse model of brain metastasis.

### Effect of WP1066 on survival and proliferation of brain metastatic cells

**Figure 2:**
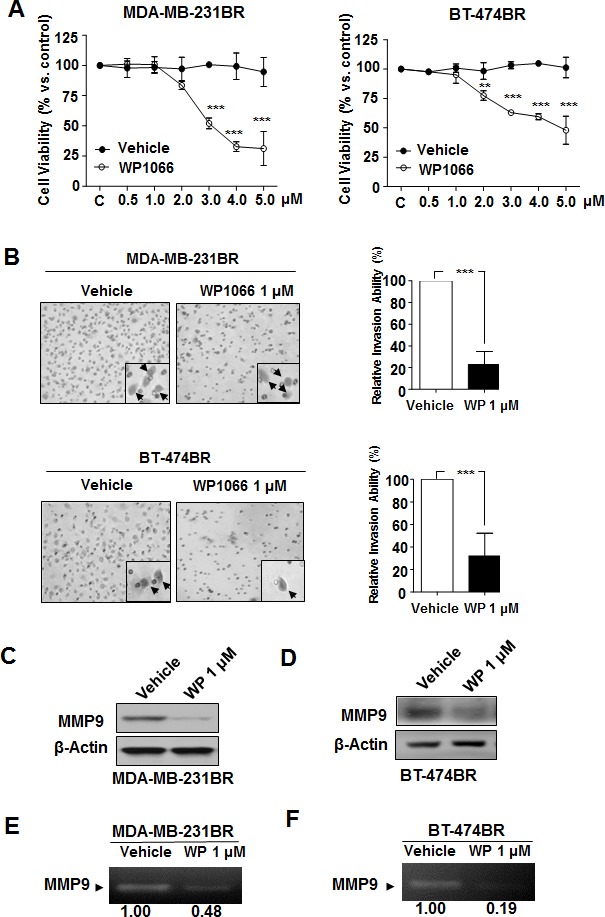
Effects of WP1066 on MDA-MB-231BR and BT-474BR cells (A) Cytotoxicity of WP1066 was measured by MTT assay. Cells were treated with DMSO or with the indicated concentrations of WP1066 for 72 hours. Values are means ± SD for triplicate experiments. **, P<0.05; ***, P<0.001. (B) Cells were treated with DMSO or WP1066 (1 μM). Then, invasiveness was determined 16 hours (MDA-MB-231BR cells) or 24 hours (BT-474BR cells) after treatment. Left panels, representative photos of the results. Right panels, quantification of the results. Each column indicates the mean ± SD from results of three different experiments. ***, P<0.001. (C, D) MMP9 and β-actin protein expression following treatment with 1 μM WP1066 analyzed by immunoblotting. (E, F) MMP9 activity in the culture media of cells treated with DMSO or WP1066 1 μM determined by gelatin zymography analysis. The MMP9 intensity was quantified by using Quantity One software from BIO-RAD. Control were given as 1.00.

To study the mechanism of inhibition of brain metastases by WP1066, we first tested the effect of WP1066 on viability of MDA-MB-231BR cells. WP1066 substantially reduced their survival in a dose-dependent manner (Fig. 2A). However, WP1066 inhibited the viability of the cells only at concentrations of 3 μM and above; WP1066 had no effect at concentrations under 2 μM (Fig. 2A). Also, WP1066 inhibited the viability of BT-474BR cells only at concentrations of 2 μM and above (Fig. 2A).

Since the concentration of WP1066 in the brain tissue was below 2 μM (Fig. 1E), we next wanted to rule out the possibility that the inhibitory effect of WP1066 against brain metastasis was caused by cytotoxicity-induced apoptosis using a TUNEL assay. The number of TUNEL-positive cells in the tumor sections was almost same in vehicle-treated mice and WP1066-treated mice (Supplemental Fig. S3), suggesting that WP1066 did not induce apoptosis of tumor cells. Thus, the inhibitory effect of WP1066 on brain metastasis was not due to cytotoxicity.

### WP1066 reduced MMP9 expression and invasion of brain metastatic cells

We sought to determine the effects of WP1066 on invasion ability of brain metastatic breast cancer cells. WP1066 (1 μM) led to a significant decrease of invasion ability of both MDA-MB-231BR and BT-474BR cells (Fig. 2B). Next, because tumor invasion is through degradation extracellular matrix and basement membrane by matrix metalloproteinases, we assessed the changes of MMP-9, the main matrix metalloproteinases in the above cell lines. MMP9 protein level and activity were decreased by 1 μM WP1066 in both cell lines (Fig. 2C-F). Consistent with the *in vitro* results, the level of MMP9 was significantly lower in brain metastases of MDA-MB-231BR cells from WP1066-treated mice than in brain metastases from control mice (Supplemental Fig. S4).

### WP1066 reduced VEGF expression and angiogenic potential of brain metastatic cells

**Figure 3:**
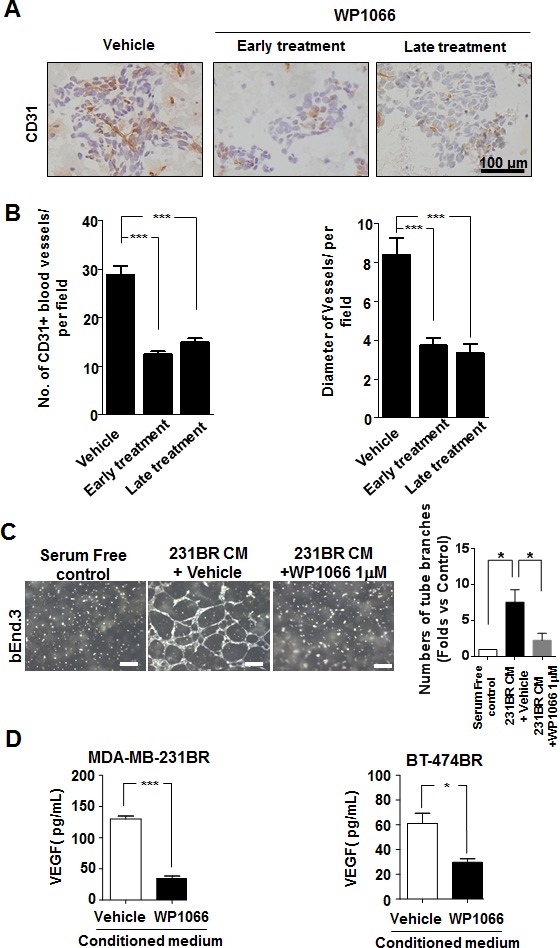
WP1066 inhibited angiogenesis of MDA-MB-231BR-cell *in vivo* and *in vitro* (A) CD31-positive blood vessels in brain sections from MDA-MB-231BR-cell-injected mice in the vehicle, early WP1066 treatment, and late WP1066 treatment groups. (B) Quantitative analysis of CD31-positive blood vessels in the brain sections of groups described in (A). Blood vessel density and average diameter were analyzed in 10 random 0.25-μm^2^ fields of each section. (C) bEnd.3 cells were treated with conditioned medium (CM) from MDA-MB-231BR cells (control), MDA-MB-231BR cells treated with vehicle, or WP1066 1 μM. The bEnd.3 cells then were subjected for tube formation assay. Tube formation in each group was photographed (left panel), and right panels show summary data (Mean ± SEM) from 3 separate experiments. *, P<0.05. (D) Quantification of VEGF concentrations by ELISA in culture media of MDA-MB-231BR and BT-474BR cells treated with DMSO or 1 μM WP1066. Each column indicates the mean ± SEM from results of five different experiments. *, P<0.05; ***, P<0.001.

Since angiogenesis is another major step of metastasis, we examined whether WP1066 would affect angiogenesis of brain metastases by assessing vascularization of brain metastases of MDA-MB-231BR cells. Brain metastases in the control group were highly vascularized whereas brain metastases in the WP1066 treatment groups had lower microvessel density (Fig. 3A and B).

Therefore, we determined the effects of WP1066 on the angiogenic ability of brain metastatic cells by conducting an endothelial cell tube formation assay with mouse brain endothelial cell line bEnd.3. The conditioned medium from WP1066-treated MDA-MB-231BR cells significantly reduced capillary tube formation, as compared with the conditioned medium from control cells (Fig. 3C). Moreover, WP1066 (1 μM) significantly reduced the expression of VEGF in MDA-MB-231BR and BT-474BR cells (Fig. 3D). Consistently, in the brain metastases, VEGF expression was prominent in the MDA-MB-231BR control group but was significantly decreased in WP1066 treatment groups (Supplemental Fig. S4).

### VEGF induced VEGFR2 expression in brain endothelial cells through Stat3 activation

**Figure 4:**
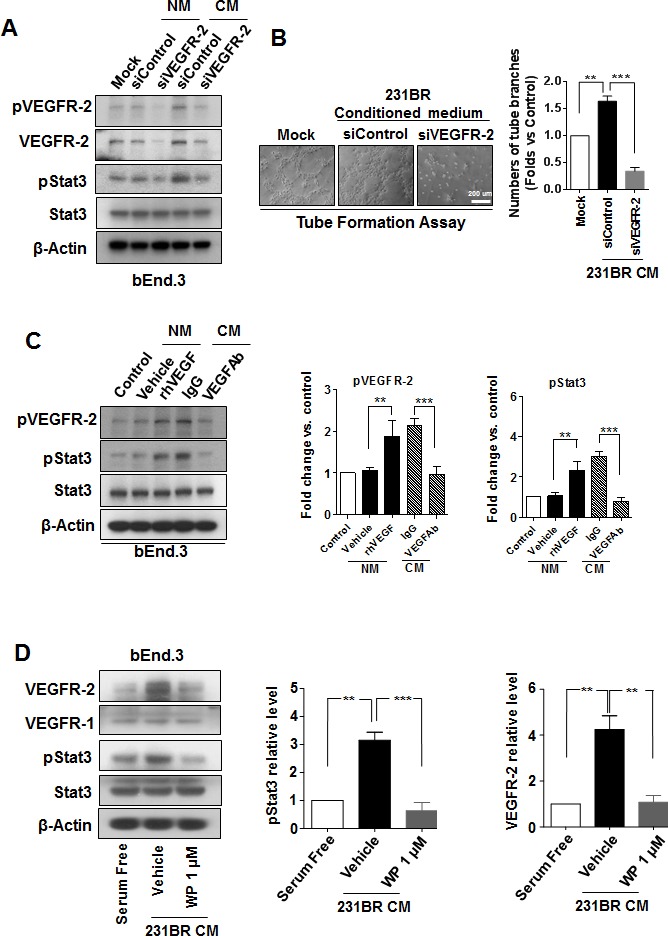
WP1066 inhibited Stat3/VEGFR-2 signaling in brain endothelial cells (A) bEnd.3 cells were transfected with 100 nmol/L siControl or siVEGFR-2 for 48 hours in normal medium (serum free) and conditioned medium of MDA-MB-231BR cells. Protein expression of VEGFR-2, pVEGFR-2, pStat3, Stat3, and β-actin was analyzed by immunoblotting. (B) bEnd.3 cells were transfected with 100 nmol/L siControl or siVEGFR-2 for 48 hours and then subjected for tube formation assay with conditioned medium (CM) of MDA-MB-231BR cells. Lefts panels show representative experiments and right panels show quantification data (mean ± SEM) from three separate experiments. **, P<0.01; ***, P<0.001. (C) bEnd.3 cells were pretreated with a recombinant human VEGF or control vehicle in normal medium or pretreated with a VEGF neutralizing antibody (VEGF-Ab) or IgG in CM of MDA-MB-231BR cells. Then protein expression of pVEGFR-2, VEGFR-2, pStat3, Stat3, and β-actin in the cells was analyzed by immunoblotting. Right panels, quantification of pStat3 and pVEGFR-2 immunoblotting results. Each column indicates the mean ± SD from results of three different experiments. **, P<0.01; ***, P<0.001. (D) bEnd.3 cells were treated with 1 μM WP1066 in CM of MDA-MB-231BR cells for 24 hours, and whole-cell lysates were subjected to western blotting for pStat3, Stat3, VEGFR-1, VEGFR-2, and β-actin (left panel). Right panels, quantification of pStat3 and VEGFR-2 immunoblotting results. Each column indicates the mean ± SD from results of three different experiments. **, P<0.01; ***, P<0.001.

The above data suggested that VEGF in the conditioned medium of brain metastatic cells might activate brain endothelial cells and cause them to undergo angiogenesis. Therefore, we examined the VEGFR2 phosphorylation in bEnd.3 cells cultured with conditioned medium of brain metastatic cells, since pVEGFR2 is a marker for endothelial cell activation [32]. The conditioned medium of MDA-MB-231BR cells induced up-regulation of pVEGFR2, as compared with serum-free culture medium (Fig. 4A). Knockdown of VEGFR2 impaired the angiogenic ability of bEnd.3 cells induced by the conditioned medium (Fig. 4A and B), indicating that VEGFR2 is indeed a marker for endothelial cell activation.

Next, we tested whether VEGF in the conditioned medium is the factor that caused VEGFR2 activation. Exogenous human VEGF increased the level of pVEGFR2 in bEnd.3 cells in serum-free medium (Fig. 4C). In contrast, a VEGF neutralizing antibody decreased the level of pVEGFR2 in bEnd.3 cells in conditioned medium (Fig. 4C). Moreover, the levels of pStat3 changed in parallel with the changes of pVEGFR2 (Fig. 4C), which is consistent with previous reports of VEGF activating Stat3 via VEGFR2 phosphorylation [33].

Surprisingly, the level of VEGFR2 was increased in bEnd.3 cells in conditioned medium of MDA-MB-231BR cells (Fig. 4D). Moreover, the level of VEGFR2, but not the level of VEGFR1, was lower in bEnd.3 cells treated with WP1066 in the conditioned medium than in bEnd.3 cells treated with control vehicle in the conditioned medium (Fig. 4D). Furthermore, the level of pStat3 was lower in in bEnd.3 cells treated with WP1066 in the conditioned medium than in bEnd.3 cells treated with control vehicle in the conditioned medium (Fig. 4D), but the total Stat3 level was not affected by WP1066 treatment (Fig. 4D). These results suggested that VEGFR2 increase induced by conditioned medium of MDA-MB-231BR cells was due to the activation of Stat3.

### Stat3 directly regulated the transcription of VEGFR2

**Figure 5:**
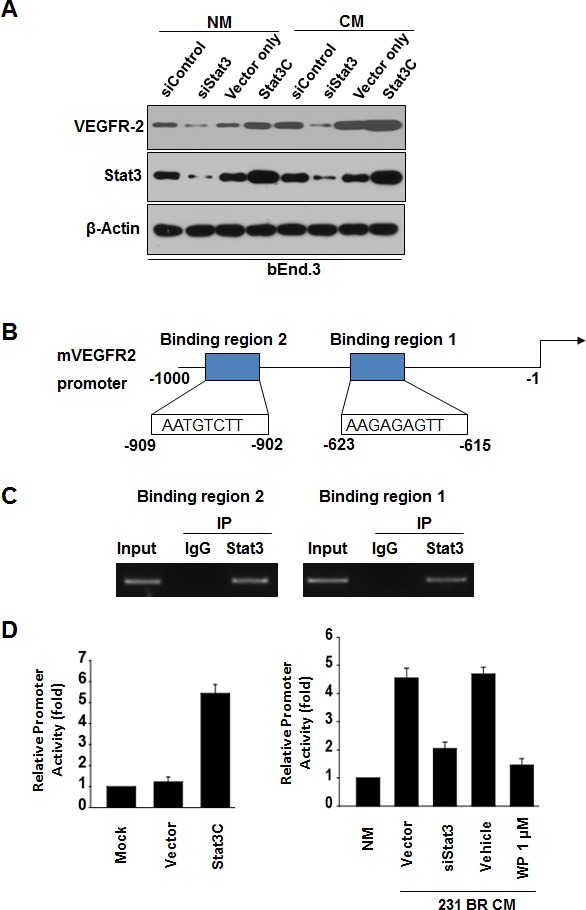
Stat3 signaling regulates VEGFR-2 expression in bEnd.3 cells (A) bEnd.3 cells were transfected with 100 nmol/L of siRNA (siControl or siStat3) or 6 μg of cDNA plasmid (vector or Stat3C) for 48 hours with serum-free normal medium (NM) or conditioned medium of MDA-MB-231BR cells (CM). Protein expression of Stat3, VEGFR-2, and β-actin was analyzed by immunoblotting. (B) Schematic structure of the murine VEGFR2 promoter with the sequences of Stat3-binding sites. (C) ChIP assays in bEnd.3 cells cultured with CM of MDA-MB-231BR cells. Chromatin fragments of the cells were immunoprecipitated with Stat3 antibody or IgG and subjected to PCR using primers for both Stat3-binding sites. (D) Stat3 transactivates VEGFR-2 promoter. Left, bEnd.3 cells were transfected with the murine VEGFR2 promoter and Stat3C or control vector (6 μg) for 48 hours in NM. Luciferase activities of the cells were then determined. Right, bEnd.3 cells were transfected with the murine VEGFR2 promoter and siStat3 or siControl (100 nmol/L) for 48 hours in CM of MDA-MB-231BR cells or treated with WP1066. bEnd.3 cells transfected with the murine VEGFR2 promoter in serum-free normal medium (NM) served as a control. Luciferase activities of the cells were then determined.

We tested whether VEGFR2 is a downstream transcriptional target of Stat3. Knockdown of Stat3 in bEnd.3 cells in serum-free medium decreased VEGFR2 expression whereas transfection of a constitutively activated mutant of Stat3 (Stat3C) plasmid led to increased VEGFR2 expression in bEnd.3 cells (Fig. 5A), indicating that Stat3 regulates the basal levels of VEGFR2 in the cells. Furthermore, knockdown of Stat3 expression in bEnd.3 cells in conditioned medium decreased VEGFR2 expression (Fig. 5A).

Next, we analyzed the sequence of the murine VEGFR-2 promoter by using the Stat3 consensus sequences AA (N4) TT and AA (N5) TT [34]. We identified two putative Stat3-binding sites in the VEGFR-2 promoter (Fig. 5B). Both of the Stat3-binding regions of the VEGFR-2 promoter bound to endogenous Stat3 protein in bEnd.3 cells by ChIP assays (Fig. 5C). Moreover, we examined whether Stat3 activation transactivates the VEGFR-2 promoter by using a murine VEGFR-2 (flk1) promoter luciferase reporter [35]. Overexpressed Stat3C upregulated VEGFR-2 promoter activity in bEnd.3 cells cultured in serum-free medium (Fig. 5D). Furthermore, VEGFR2 promoter activity was increased in bEnd.3 cells in conditioned medium of MDA-MB-231BR cells (Fig. 5D), whereas VEGFR2 promoter activity was decreased in bEnd.3 cells with Stat3 siRNA or with conditioned medium of MDA-MB-231BR cells treated with WP1066 (Fig. 5D). Consistent with the *in vitro* results, VEGFR-2 expression in brain metastases was significantly reduced by WP1066 treatment in mice (Supplemental Fig. S4). Collectively, these results indicated that VEGFR2 is a direct transcriptional target of Stat3 and that WP1066 downregulates VEGFR-2 expression by inhibition of Stat3.

### WP1066 inhibited the activation hence migration and invasion of brain endothelial cells induced by brain metastatic breast cancer cells

**Figure 6:**
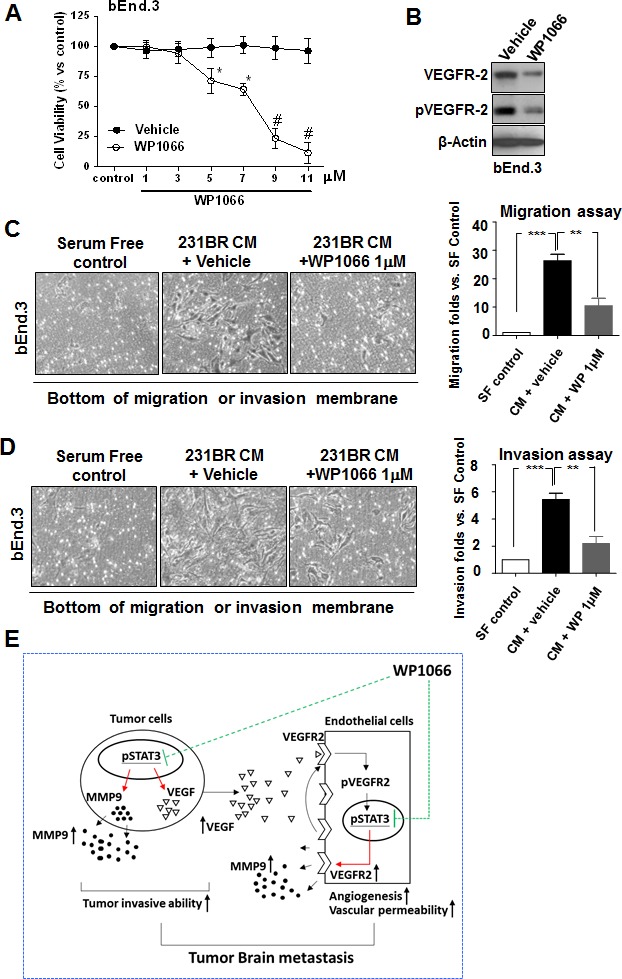
WP1066 inhibits the invasion and migration ability of bEnd.3 cells (A) Cytotoxicity of WP1066 in bEnd.3 cells by MTT assay. bEnd.3 cells were treated with various concentrations of WP1066 or with DMSO for 72 hours. Values are means ± SD for triplicate experiments. *, P<0.1; #, P<0.001. (B) bEnd.3 cells were treated with vehicle or WP1066 1 μM in CM of MDA-MB-231BR cells. Protein expression of VEGFR-2, pVEGFR-2, and β-actin was analyzed by immunoblotting. (C) bEnd.3 cells were cultured with serum-free medium or CM from MDA-MB-231BR cells treated with vehicle or WP1066 1 μM. Then migration ability was determined using the migration assay at 12 hours. Left panels, representative photos of the results. Right panel, quantification of results. Each column indicates the mean ± SD from results of three different experiments. **, P<0.01; ***, P<0.001. (D) bEnd.3 cells were cultured medium as described in (C). Then invasiveness was determined using the invasion assay at 16 hours. Left panels, representative photos of the results. Right panel, quantification of results. Each column indicates the mean ± SD from results of three different experiments. **, P<0.01; ***, P<0.001. (E) Stat3 regulates breast cancer cell brain metastasis by affecting interaction between tumor cells and endothelial cells and tumor cell invasive. Activated Stat3 in tumor cells upregulates the expression of VEGF that induces the phosphorylation of VEGFR2 in endothelial cells. The phosphorylated VEGFR2 leads to phosphorylation hence activation of Stat3 in endothelial cells. Then the activated Stat3 directly upregulates VEGFR2 expression in endothelial cells, which leads to further activation of VEGFR2 in the cells, increasing their infiltration and angiogenesis. Therefore, Stat3 activation orchestrates the interaction between endothelial and tumor cells. Stat3 also directly regulates MMP-9 expression hence breast cancer cell and endothelial invasion. WP1066 can effectively inhibit tumor angiogenesis and invasion thus brain metastasis mediated by Stat3.

The above results indicated that Stat3 activity regulated VEGFR-2 in brain endothelial cells and hence regulated their activation, thus, we examined whether WP1066 has direct cytotoxic effects on brain endothelial cells. WP1066 at low concentrations (1-3 μM) was not cytotoxic to bEnd.3 cells (Fig. 6A). However, a low concentration of WP1066 (1 μM) inhibited the activation of brain endothelial cells induced by brain metastatic breast cancer cells (Fig. 6B).

Since migration and invasion of brain endothelial cells require the activation of the cells, we examined whether conditioned medium of MDA-MB-231BR cells could induce migration and invasion of brain endothelial cells. Migration and invasion of bEnd.3 cells in conditioned medium of MDA-MB-231BR cells was increased compared to that in serum-free medium (Fig. 6C and D). In contrast, migration and invasion of bEnd.3 cells in conditioned medium from WP1066-treated MDA-MB-231BR cells was decreased (Fig. 6C and D). These results indicated that WP1066 inhibited the invasion and migration of brain endothelial cells induced by brain metastatic breast cancer cells.

## DISCUSSION

In the present study, we sought to determine the role of Stat3 in the interaction between tumor cells and endothelial cells in breast cancer brain metastases. Our novel clinical and mechanistic evidence strongly suggests that activated Stat3 binds directly to the VEGFR2 promoter and increases its transcription (Fig. 6E). Conversely, VEGFR2 positively regulates activation of Stat3 and invasion of brain endothelial cells (Fig. 6E). Moreover, suppression of Stat3 activation by the small molecular inhibitor WP1066 inhibited the activation of brain endothelial cells and brain metastasis of breast cancer cells in an animal model, presumably by inhibition of the transcription of Stat3-target genes including VEGFR2 and MMP-9 in both endothelial cells and tumor cells (Fig. 6E).

The growth and propagation of brain metastases depend on the establishment of an adequate blood supply—i.e., angiogenesis. The angiogenic phenotype of brain metastases have been shown to be associated with an increase in the production of proangiogenic molecules by the tumor cells and brain environments [2-5]. Previous studies have established that VEGF is involved in breast cancer brain metastasis [36]. However, the function of VEGF depends on the activation of receptors, including VEGFR2.

VEGFR2 is mainly expressed in endothelial cells [11]. VEGFR2 activation induces cell proliferation, migration, and tube formation of endothelial cells [37]. VEGFR2 is also expressed in brain metastasis-associated endothelial cells [38]. Targeting VEGFR2 with an anti-VEGFR2 antibody in combination with a HER2 inhibitor significantly slowed breast cancer growth in the brain, resulting in a striking survival benefit [38]. Furthermore, we found that MDA-MB-231BR cells also express VEGFR2 (data not shown). Therefore, VEGFR2 appears to be a critical target for the suppression of brain metastasis. However, the mechanisms for the overexpression and activation of VEGFR2 in breast cancer brain metastasis are unclear.

The transcription factors Sp1, HoxB5, and GATA-2 have been implicated in regulation of VEGFR2 expression via its promoter in other diseases [39-41] whereas the transcription factor FoxC2 regulates VEGFR2 expression through binding of VEGFR-2 enhancer [14]. The current study is the first to show that Stat3 regulates the transcription of VEGFR2. Our data adds a new mechanism to the body of knowledge on the transcriptional regulation of VEGFR2 expression by providing evidence that VEGFR2 is a direct transcriptional target of Stat3 and that Stat3 critically controls constitutive and inducible VEGFR2 expression in endothelial cells. More importantly, our study indicates that Stat3 activation orchestrates the interaction between endothelial cells and tumor cells, which is known to be crucial for tumor growth and metastasis [6, 42-45]. Stat3 upregulates VEGF expression in tumor cells and that VEGF induces the activation of VEGFR2 in endothelial cells. The activated VEGFR2 then activates Stat3 in endothelial cells, which leads to VEGFR2 upregulation and further activation. This Stat3-orchestrated interaction between endothelial and tumor cells resulted in a number of cellular activities involved in angiogenesis, including endothelial cell migration, invasion and tube formation. Blocking of Stat3 by WP1066 resulted in reduced VEGFR-induced signaling and angiogenesis, which further supports the role of Stat3 in the interaction of tumor cells and endothelial cells.

Our data validate, in a preclinical model, the efficacy of WP1066 for the inhibition of brain metastasis by human breast cancer cells. The inhibition of brain metastasis by WP1066 was paralleled by reduced pStat3 staining in the remaining lesions *in vivo*, confirming that the drug affected its intended target. Taken together, our findings indicate that blocking Stat3 activity with WP1066 may have promise in the treatment of brain metastasis of breast cancer through targeting both tumor cells and tumor-associated endothelial cells.

## MATERIALS AND METHODS

### Cell lines and culture conditions

MDA-MB-231BR and BT-474BR “brain-seeking” cell lines were described previously [26, 28]. Mouse brain endothelial cell line bEnd.3 was purchased from American Type Culture Collection. All cell lines were maintained in Eagle's MEM supplemented with 10% fetal bovine serum, sodium pyruvate, nonessential amino acids, L-glutamine, and a twofold vitamin solution (Flow Laboratories).

### Human tissue specimens

Tissue from human breast invasive ductal carcinoma and breast cancer brain metastases were coded without any patient identifiers. The use of the tissue was approved by the Institutional Review Board of The University of Texas MD Anderson Cancer Center. Archival paraffin-embedded specimens from 90 invasive ductal carcinomas and 89 breast cancer brain metastases were used in the study.

### Reagents

WP1066 was synthesized at The University of Texas MD Anderson Cancer Center and dissolved in dimethyl sulfoxide (DMSO) for *in vitro* studies. For *in vivo* experiments, WP1066 was administered via intraperitoneal injection in a vehicle of DMSO/polyethylene glycol (PEG) 300 (1:4 ratio) (Sigma). Recombinant human VEGF and neutralizing VEGF antibody were from R&D Systems. SMARTpool siRNA duplexes specific for Stat3 and VEGFR2 and a nontargeting siRNA (siControl) were purchased from Dharmacon.

### Experimental brain metastasis model

Female athymic BALB/c nude mice were purchased from the National Cancer Institute (Frederick, MD). The mouse experiments were conducted under a protocol approved by the Institute Animal Care and Use Committee of MD Anderson Cancer Center. Mice were inoculated with 5 × 10^5^ MDA-MB-231BR cells in the heart's left ventricle. A 100-μL intraperitoneal injection of WP1066 (40 mg/kg in DMSO/PEG 300 vehicle) or vehicle alone was administrated every other day. The Size of metastases was measured by 16mm^2^ ocular grid. The mean numbers of large metastases (>300 μM on the long axis) and micrometastases (smaller than large metastases) were determined by counting the number of metastases from one hemisphere of the brain as described previously [4].

### Preparation of tumor cell conditioned medium

MDA-MB-231BR or BT-474BR (5×10^6^) were seeded in 10cm plates and incubated overnight at 37^o^C. The cells were washed twice with Hanks' balanced salt solution and cultured for an additional 24 h in serum-free medium, and the supernatants were collected as conditional medium. Also, MDA-MB-231BR or BT-474BR cells were treated with DMSO or WP1066 (1 μM) in serum-free culture medium for 24 hours, and the supernatants were collected as conditional medium.

### Western blot analysis

Standard western blotting of whole cell lysates was performed with antibodies for Stat3, Stat3 phosphorylated at Tyr705 (Cell Signaling Technologies), VEGF, VEGFR2, and phosphorylated VEGFR2 (Santa Cruz Biotechnology). β-actin (Sigma) was used as a control for loading.

### Statistical analysis

The significance of the data from patient specimens was determined by χ^2^ test. The significance of the *in vitro* data was determined by Student's *t*-test (two-tailed), whereas the significance of the *in vivo* data was determined by using one-way analysis of variance.
